# Joint effects of mitochondrial DNA 5178 C/A polymorphism and coffee consumption or alcohol consumption on clustering of cardiovascular risk factors in middle-aged Japanese men: a cross-sectional study

**DOI:** 10.1186/2251-6581-13-4

**Published:** 2014-01-06

**Authors:** Taku Ito, Akatsuki Kokaze, Mamoru Ishikawa, Naomi Matsunaga, Kanae Karita, Masao Yoshida, Tadahiro Ohtsu, Hirotaka Ochiai, Takako Shirasawa, Hinako Nanri, Hiromi Hoshino, Yutaka Takashima

**Affiliations:** 1 Department of Public Health, Showa University School of Medicine, 1-5-8 Hatanodai, Shinagawa-ku, Tokyo 142-8555, Japan; 2 Department of Public Health, Kyorin University School of Medicine, 6-20-2 Shinkawa, Mitaka-shi, Tokyo 181-8611, Japan; 3 Mito Red Cross Hospital, 3-12-48 Sannomaru, Mito-shi, Ibaraki 310-0011, Japan

**Keywords:** Alcohol consumption, Cardiovascular risk factor, Coffee consumption, Mitochondrial DNA polymorphism, Personalized prevention

## Abstract

**Background:**

Longevity-associated mitochondrial DNA 5178 (Mt5178) C/A reportedly modulates the effects of coffee consumption on the risk of hypertension, dyslipidemia and abnormal glucose tolerance, and those of alcohol consumption on the risk of hypertension, dyslipidemia and hyperuricemia in middle-aged Japanese men. However, there has been no research examining whether Mt5178 C/A polymorphism influences the effects of coffee consumption or alcohol consumption on the clustering of cardiovascular risk factors (CRFs).

**Methods:**

A total of 332 male subjects (mean age ± SD, 52.8 ± 7.8 years) were selected from among individuals visiting the hospital for regular medical check-ups. After Mt5178 C/A genotyping, a cross-sectional study assessing the joint effects of Mt5178 C/A polymorphism and coffee consumption or alcohol consumption on the clustering of CRFs, namely hypertension, abnormal glucose tolerance, hyper-low-density lipoprotein cholesterolemia, hypo-high density lipoprotein cholesterolemia, hypertriglyceridemia and hyperuricemia, was then conducted.

**Results:**

After adjustment for confounding factors, significant and negative associations were observed between coffee consumption and clustering of ≥2 CRFs in subjects with Mt5178C. The adjusted odds ratio (OR) for the clustering of ≥2 or ≥3 CRFs was significantly lower in subjects who consumed 1–3 cups of coffee per day than in those who consumed <1 cup of coffee per day (OR = 0.496, 95% confidence interval (CI): 0.249–0.989, and OR = 0.369, 95% CI: 0.165–0.826, respectively). On the other hand, after adjustment, positive associations between coffee consumption and clustering of ≥2 CRFs were observed in subjects with Mt5178A. However, these associations did not reach a significant level. For Mt5178C genotypic men, the adjusted OR for the clustering of ≥2 or ≥3 CRFs was significantly higher in daily drinkers than in occasional drinkers (OR = 2.737, 95% CI: 1.361–5.502, and OR = 3.024, 95% CI: 1.269–7.210, respectively). On the other hand, the association between Mt5178A genotype and the clustering of ≥2 or ≥3 CRFs did not appear to depend on alcohol consumption.

**Conclusions:**

The present results suggest that Mt5178 C/A polymorphism modifies the effects of coffee consumption or alcohol consumption on the clustering of CRFs in middle-aged Japanese men.

## Introduction

Cardiovascular disease (CVD) is public health issue of great importance. From the standpoint of CVD prevention, it is absolutely essential to reduce the number of cardiovascular risk factors (CRFs), namely, hypertension, dyslipidemia, abnormal glucose tolerance (AGT) and hyperuricemia [1,2]. Several CRFs evidently provoke mitochondrial damage and dysfunction [3,4]. Mitochondrial dysfunction results in impairment of oxidative phosphorylation, and increases in both reactive oxygen species (ROS) and calcium dysregulation. These mitochondria-related adverse effects promote apoptosis, cellular senescence, and eventually, atherosclerosis [4].

Mitochondrial DNA 5178 (Mt5178) C/A polymorphism, also known as NADH dehydrogenase subunit-2 237 (ND2-237) Leu/Met, is a longevity-associated mitochondrial DNA polymorphism [5,6]. The frequency of the Mt5178A genotype is significantly higher in Japanese centenarians than in the general population. Japanese individuals with Mt5178A are more resistant to atherosclerotic diseases, such as myocardial infarction [7,8] and cerebrovascular disorders [9], than those with Mt5178C. Our previous studies have reported the joint effects of Mt5178 C/A polymorphism and coffee consumption on the risk of hypertension [10], dyslipidemia [11], and AGT [12] in middle-aged Japanese men. We have also reported the combined effects of Mt5178 C/A polymorphism and alcohol consumption on the risk of hypertension [13], dyslipidemia [14], and hyperuricemia [15]. However, an examination of whether Mt5178 C/A polymorphism influences the effects of coffee and alcohol consumption on clustering of CRFs has yet to be performed.

The objective of this study was to investigate whether there is a joint effect of Mt5178 C/A polymorphism and coffee consumption or alcohol consumption on the clustering of CRFs in middle-aged Japanese male subjects.

## Materials and methods

### Study subjects

Participants were recruited from among individuals visiting the Mito Red Cross Hospital for regular medical check-ups between August 1999 and August 2000. This study was conducted in accordance with the Declaration of Helsinki and approved by the Ethics Committee of the Kyorin University School of Medicine. Written informed consent was obtained from all 602 volunteers before participation. Because the number of women was insufficient for classification into groups based on Mt5178 C/A genotype and coffee consumption or alcohol consumption habits, they were excluded. Individuals not receiving 75-g oral glucose tolerance test were also excluded. Hyper-low-density lipoprotein (LDL) cholesterolemia, hypo-high-density lipoprotein (HDL) cholesterolemia, and hypertriglyceridemia were regarded as three different CRFs. Therefore, due to a lack of information on the types of lipid-lowering drugs being used, those taking any lipid-lowering medications were excluded. Those with unclear data were also excluded. Thus, the subjects in this study comprised a total of 332 Japanese men (mean age ± SD, 52.8 ± 7.8 years).

### Clinical characteristics of subjects

Determination of blood chemical and physical data was conducted as described previously [16]. Serum total cholesterol levels, serum HDL cholesterol levels, serum triglyceride levels, fasting plasma glucose (FPG) levels and serum uric acid levels were measured using standard laboratory techniques. Serum LDL cholesterol levels were calculated by Friedewald’s formula [17]. For both systolic blood pressure (SBP) and diastolic blood pressure (DBP), the mean of two consecutive values measured by physicians was used. Body mass index (BMI) was defined as the ratio of subject weight (kg) to the square of subject height (m). Hypertension was defined as SBP ≥140 mmHg and/or DBP ≥90 mmHg and/or antihypertensive drug treatment. AGT was defined as FPG ≥110 mg/dl and/or 2-h plasma glucose on the 75-g oral glucose tolerance test ≥140 mg/dl, or diagnosed with diabetes. According to the Japan Atherosclerosis Society guidelines for prevention of atherosclerotic cardiovascular diseases [18], hyper-LDL cholesterolemia was defined as serum LDL cholesterol ≥140 mg/dl, hypo-HDL cholesterolemia was defined as serum HDL cholesterol <40 mg/dl, and hypertriglyceridemia was defined as serum triglyceride ≥150 mg/dl. Similar to other studies on clustering of CRFs [19,20], hyperuricemia was defined as serum uric acid ≥7.0 mg/dl. The number of CRFs was calculated by adding the number of these components, namely hypertension, AGT, hyper-LDL cholesterolemia, hypo-HDL cholesterolemia, hypertriglyceridemia and hyperuricemia. Therefore, the number of CRFs varied from 0 to 6 in integral values. A questionnaire survey of coffee intake, alcohol consumption, and habitual smoking was also conducted. Coffee consumption was classified based on number of cups of coffee per day (<1 cup per day; 1–3 cups per day; and ≥4 cups per day). Alcohol consumption was classified based on drinking frequency (daily drinkers; occasional drinkers, which include those who drink several times per week or per month; and non- or ex-drinkers). Smoking status was classified based on number of cigarettes smoked per day (never- or ex-smokers; 1–20 cigarettes smoked per day; and >20 cigarettes smoked per day).

### Genotyping

DNA was extracted from white blood cells using the DNA Extractor WB kit (Wako Pure Chemical Industries, Japan). Mt5178 C/A polymorphism was detected by polymerase chain reaction (PCR) and digestion with *Alu*I restriction enzyme. The sequence of primers was: forward 5′-CTTAGCATACTCCTCAATTACCC-3′, reverse 5′-GTGAATTCTTCGATAATGGCCCA-3′. PCR was performed with 50 ng of genomic DNA in buffer containing 0.2 μmol/l each primer, 1.25 mmol/l dNTPs, 1.5 mmol/l MgCl_2_, and 1 U of Taq DNA polymerase. After an initial denaturation at 94°C for 5 min, PCR was conducted through 40 cycles in the following steps: denaturation at 94°C for 30 s, annealing at 60°C for 60 s, and polymerase extension at 72°C for 90 s. After cycling, a final extension at 72°C for 10 min was performed. PCR products were digested with *Alu*I restriction enzyme (Nippon Gene, Japan) at 37°C overnight and electrophoresed on 1.5% agarose gels stained with ethidium bromide for visualization under ultraviolet light. The absence of an *Alu*I site was designated as Mt5178A (279-bp fragment), and the presence of this restriction site was designated as Mt5178C (175-bp and 104-bp fragments).

### Statistical analyses

Statistical analyses were performed using SAS statistical software for Windows (version 9.3, SAS Institute Inc., Cary, NC). Multiple logistic regression analysis was used to calculate the odds ratio (OR) for the clustering of CRFs, namely more than 2 or 3 CRF components. For multiple logistic regression analysis, coffee consumption (<1 cup per day = 1, 1–3 cups per day = 2, ≥4 cups per day = 3), alcohol consumption (non- or ex-drinkers = 0, occasional drinkers, which include those who drink several times per week or per month = 1, daily drinkers = 2), and habitual smoking (never- or ex-smokers = 0, 1–20 cigarettes smoked per day = 1, >20 cigarettes smoked per day = 2) were numerically coded. Based on previous reports [10-15], subjects consuming <1 cup of coffee per day and occasional drinkers were used for reference. Differences with *P* values less than 0.05 were considered to be statistically significant.

## Results

No significant differences were observed between the Mt5178C and Mt5178A genotypes in biophysical or biochemical characteristics or in frequencies of CRFs, namely hypertension, AGT, hyper-LDL cholesterolemia, hypo-HDL cholesterolemia, hypertriglyceridemia and hyperuricemia (Table 1).

**Table 1:** Clinical characteristics of study subjects by Mt5178 C/A genotype

	**Mt5178C**	**Mt5178A**	***P *** **value**
	**N = 197**	**N = 135**	
Age (y)	53.0 ± 7.9	52.6 ± 7.8	0.602
Body mass index (kg/m^2^)	23.2 ± 2.6	23.6 ± 2.5	0.165
Systolic blood pressure (mmHg)	125.0 ± 15.4	125.2 ± 13.8	0.872
Diastolic blood pressure (mmHg)	72.8 ± 10.1	72.5 ± 8.5	0.788
Total cholesterol (mg/dl)	204.9 ± 35.8	204.8 ± 32.0	0.982
LDL cholesterol (mg/dl)	121.6 ± 36.7	120.6 ± 31.3	0.796
HDL cholesterol (mg/dl)	54.6 ± 13.6	56.2 ± 16.1	0.344
Triglyceride (mg/dl)	143.1 ± 97.5	139.9 ± 90.2	0.764
Fasting plasma glucose (mg/dl)	96.4 ± 8.8	97.1 ± 9.2	0.517
2-h plasma glucose on 75-g oral glucose tolerance test (mg/dl)	128.3 ± 43.3	124.6 ± 37.9	0.433
Uric acid (mg/dl)	5.99 ± 1.24	5.96 ± 1.22	0.798
Coffee consumption (<1 cup per day/1-3 cups per day/≥ 4 cups per day) (%)	40.1/48.7/11.2	39.3/48.9/11.8	0.977
Alcohol consumption (non- or ex-/occasional/daily) (%)	19.3/36.5/44.2	12.6/38.5/48.9	0.267
Smoking status (never- or ex-/1–20 cigarettes per day/>20 cigarettes per day) (%)	57.4/29.4/13.2	58.5/27.4/14.1	0.914
Hypertension (%)	28.4	21.5	0.154
Abnormal glucose tolerance (%)	33.5	31.1	0.648
Hyper-LDL cholesterolemia (%)	27.4	28.9	0.768
Hypo-HDL cholesterolemia (%)	11.2	11.1	0.987
Hypertriglyceridemia (%)	32.0	28.2	0.456
Hyperuricemia (%)	20.8	19.3	0.729

LDL; low-density lipoprotein, HDL; high-density lipoprotein. Age, body mass index, systolic blood pressure, diastolic blood pressure, serum total cholesterol levels, serum LDL cholesterol levels, serum HDL cholesterol levels, serum triglyceride levels, fasting plasma glucose levels, 2-h plasma glucose levels on 75-g oral glucose tolerance test and serum uric acid levels are given as means ± S.D. *P* values were calculated by Student’s t-test. For alcohol consumption, coffee consumption, smoking status, hypertension, abnormal glucose tolerance, hyper-LDL cholesterolemia, hypo-HDL cholesterolemia, hypertriglyceridemia and hyperuricemia, *P* values were calculated by chi-squared test. All *P* values indicate significance of differences between Mt5178C and Mt5178A.

**Figure 1:**
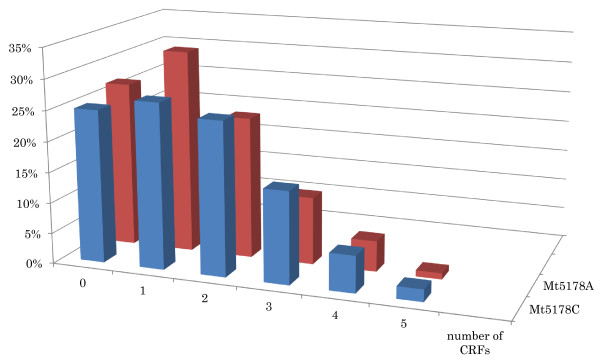
Distribution of number of cardiovascular risk factors (CRFs) between the Mt5178C and Mt5178A genotypes.

Among subjects with Mt5178C, frequencies of those with 0, 1, 2, 3, 4 and 5 CRFs were 25.4%, 26.9%, 25.4%, 15.2%, 5.6% and 1.5%, respectively (Figure 1). Among subjects with Mt5178A, frequencies of those with 0, 1, 2, 3, 4 and 5 CRFs were 26.7%, 32.6%, 22.9%, 11.1%, 5.2% and 1.5%, respectively. No subjects had 6 CRFs in either the Mt5178C or the Mt5178A genotype. A chi-square test did not show a statistically significant difference between the Mt5178C and Mt5178A genotypes in the frequencies of subjects with number of CRFs (*P* = 0.827).

After adjustment for age and BMI or for age, BMI, habitual smoking and alcohol consumption, significant and negative associations were observed between coffee consumption and the clustering of ≥2 CRFs in subjects with Mt5178C (*P* for trend = 0.030 and *P* for trend = 0.014, respectively) (Table 2). Although the crude OR for the clustering of ≥2 CRFs was not significant, after adjustment for age, BMI, habitual smoking and alcohol consumption, the OR for the clustering of ≥2 CRFs was significantly lower both in subjects who consumed 1–3 cups of coffee per day and in those who consumed ≥4 cups of coffee per day, as compared to those who consumed <1 cup of coffee per day (OR = 0.496, 95% confidence interval (CI): 0.249-0.989, *P* = 0.047 and OR = 0.259, 95% CI: 0.074-0.901, *P* = 0.034, respectively). These results indicate that the covariates, namely age, BMI, habitual smoking and alcohol consumption, were potential confounding factors. Moreover, the crude OR for clustering of ≥3 CRFs was significantly lower in subjects who consumed 1–3 cups of coffee per day than in those who consumed <1 cup of coffee per day (OR = 0.451, 95% CI: 0.216–0.940, *P* = 0.034). After adjustment for age and BMI or for age, BMI, habitual smoking and alcohol consumption, a significant OR remained (OR = 0.376, 95% CI: 0.171–0.824, *P* = 0.015 and OR = 0.369, 95% CI: 0.165–0.826, *P* = 0.015, respectively). However, negative associations between coffee consumption and the clustering of ≥3 CRFs did not reach a significant level for either adjustment (*P* for trend = 0.097 or *P* for trend = 0.069, respectively). On the other hand, after both adjustments, positive associations between coffee consumption and the clustering of ≥2 CRFs were observed in subjects with Mt5178A; however, these associations did not reach a level of significance (*P* for trend = 0.059 or *P* for trend = 0.068, respectively).

**Table 2:** **Odds ratios (ORs) and 95**% **confidence intervals (CIs) for clustering of CRFs by Mt5178 C/A genotype and coffee consumption**

**Genotype and coffee consumption**	**Frequency**		**OR (95% CI)**	**Adjusted OR† (95% CI)**	**Adjusted OR‡ (95% CI)**
	Number of CRF components				
	<2	≥2			
Mt5178C					
<1 cup per day (%)	37 (46.8)	42 (53.2)	1 (reference)	1 (reference)	1 (reference)
1-3 cups per day (%)	53 (55.2)	43 (44.8)	0.715 (0.393-1.299)	0.536 (0.277-1.037)	0.496 (0.249-0.989)*
≥4 cups per day (%)	13 (59.1)	9 (40.9)	0.610 (0.234-1.590)	0.334 (0.105-1.066)	0.259 (0.074-0.901)*
			*P* for trend = 0.208	*P* for trend = 0.030	*P* for trend = 0.014
Mt5178A					
<1 cup per day (%)	33 (62.3)	20 (37.7)	1 (reference)	1 (reference)	1 (reference)
1-3 cups per day (%)	39 (59.1)	27 (40.9)	1.142 (0.544-2.397)	1.626 (0.694-3.810)	1.596 (0.638-3.979)
≥4 cups per day (%)	8 (50.0)	8 (50.0)	1.650 (0.535-5.090)	2.989 (0.811-11.01)	3.283 (0.743-14.51)
			*P* for trend = 0.416	*P* for trend = 0.059	*P* for trend = 0.068
	Number of CRF components				
	<3	≥3			
Mt5178C					
<1 cup per day (%)	56 (70.9)	23 (29.1)	1 (reference)	1 (reference)	1 (reference)
1-3 cups per day (%)	81 (84.4)	15 (15.6)	0.451 (0.216-0.940)*	0.376 (0.171-0.824)*	0.369 (0.165-0.826)*
≥4 cups per day (%)	16 (72.7)	6 (27.3)	0.913 (0.317-2.626)	0.487 (0.136-1.746)	0.402 (0.103-1.568)
			*P* for trend = 0.267	*P* for trend = 0.097	*P* for trend = 0.069
Mt5178A					
<1 cup per day (%)	43 (81.1)	10 (18.9)	1 (reference)	1 (reference)	1 (reference)
1-3 cups per day (%)	55 (83.3)	11 (16.7)	0.860 (0.334-2.212)	0.938 (0.352-2.495)	0.762 (0.268-2.168)
≥4 cups per day (%)	13 (81.3)	3 (18.7)	0.992 (0.237-4.153)	1.485 (0.313-7.043)	0.735 (0.124-4.359)
			*P* for trend = 0.886	*P* for trend = 0.840	*P* for trend = 0.730

CRF; cardiovascular risk factor. †OR adjusted for age and body mass index. ‡OR adjusted for age, body mass index, habitual smoking and habitual alcohol consumption.

*P < 0.05.

For Mt5178C genotypic men, the crude OR for the clustering of ≥2 CRFs was significantly higher in daily drinkers than in occasional drinkers (OR = 2.391, 95% CI: 1.258–4.542, *P* = 0.008) (Table 3). After adjustment for age and BMI or for age, BMI, habitual smoking and coffee consumption, a significant OR remained (OR = 2.539, 95% CI: 1.293–4.984, *P* = 0.007 and OR = 2.737, 95% CI: 1.361–5.502, *P* = 0.005, respectively). Moreover, the crude OR for the clustering of ≥3 CRFs was significantly higher in daily drinkers than in occasional drinkers (OR = 2.984, 95% CI: 1.293–6.882, *P* = 0.010). After both adjustments, a significant OR also remained (OR = 2.918, 95% CI: 1.241–6.861, *P* = 0.014 and OR = 3.024, 95% CI: 1.269–7.210, *P* = 0.013, respectively). On the other hand, the association between Mt5178A genotype and the clustering of ≥2 or ≥3 CRFs does not appear to depend on alcohol consumption.

**Table 3:** **Odds ratios (ORs) and 95**% **confidence intervals (CIs) for clustering of CRFs by Mt5178 C/A genotype and alcohol consumption**

**Genotype and alcohol consumption**	**Frequency**		**OR (95% CI)**	**Adjusted OR† (95% CI)**	**Adjusted OR‡ (95% CI)**
	Number of CRF components				
	<2	≥2			
Mt5178C					
Non- or ex-drinkers (%)	20 (52.6)	18 (47.4)	1.592 (0.717-3.537)	2.142 (0.888-5.167)	2.212 (0.855-5.723)
Occasional drinkers (%)	46 (63.9)	26 (36.1)	1 (reference)	1 (reference)	1 (reference)
Daily drinkers (%)	37 (42.5)	50 (57.5)	2.391 (1.258-4.542)**	2.539 (1.293-4.984)**	2.737 (1.361-5.502)**
			*P* for trend = 0.106	*P* for trend = 0.168	*P* for trend = 0.172
Mt5178A					
Non- or ex-drinkers (%)	9 (52.9)	8 (47.1)	1.679 (0.553-5.098)	1.454 (0.433-4.876)	1.141 (0.352-5.677)
Occasional drinkers (%)	34 (65.4)	18 (34.6)	1 (reference)	1 (reference)	1 (reference)
Daily drinkers (%)	37 (56.1)	29 (43.9)	1.480 (0.699-3.135)	1.656 (0.736-3.725)	1.623 (0.681-3.872)
			*P* for trend = 0.305	*P* for trend = 0.223	*P* for trend = 0.275
	Number of CRF components				
	<3	≥3			
Mt5178C					
Non- or ex-drinkers (%)	29 (76.3)	9 (23.7)	2.172 (0.781-6.044)	2.525 (0.851-7.492)	2.611 (0.808-8.443)
Occasional drinkers (%)	63 (87.5)	9 (12.5)	1 (reference)	1 (reference)	1 (reference)
Daily drinkers (%)	61 (70.1)	26 (29.9)	2.984 (1.293-6.882)*	2.918 (1.241-6.861)*	3.024 (1.269-7.210)*
			*P* for trend = 0.174	*P* for trend = 0.218	*P* for trend = 0.259
Mt5178A					
Non- or ex-drinkers (%)	13 (76.5)	4 (23.5)	1.470 (0.388-5.566)	1.301 (0.330-5.132)	1.720 (0.394-7.519)
Occasional drinkers (%)	43 (82.7)	9 (17.3)	1 (reference)	1 (reference)	1 (reference)
Daily drinkers (%)	55 (83.3)	11 (16.7)	0.955 (0.363-2.513)	0.978 (0.364-2.632)	1.023 (0.368-2.844)
			*P* for trend = 0.927	*P* for trend = 0.965	*P* for trend = 0.966

CRF; cardiovascular risk factor. †OR adjusted for age and body mass index. ‡OR adjusted for age, body mass index, habitual smoking and coffee consumption.

*P < 0.05, **P < 0.001.

## Discussion

The present study shows a novel gene-environment interaction on the clustering of CRF components such as hypertension, hypertriglycemia, hypo-HDL cholesterolemia, hyper-LDL cholesterolemia, AGT and hyperuricemia. Longevity-associated Mt5178 C/A polymorphism and coffee or alcohol consumption may combine to modify the risk of 2 or 3 CRFs in middle-aged Japanese men. For men with Mt5178C, coffee consumption may decrease and daily alcohol consumption may increase the clustering of CRFs. However, for those with Mt5178A, coffee or alcohol consumption appears to have no influence.

Several clinical epidemiological studies have reported that Japanese individuals with Mt5178C are more susceptible to cardiovascular diseases than those with Mt5178A [7-9]. Considering the results of this investigation, for Mt5178C genotypic men, consumption of more than a cup of coffee per day and abstinence of daily alcohol consumption are recommended. However, this study focused on one single nucleotide polymorphism, namely Mt5178 C/A polymorphism. Therefore, to establish the individualized prevention for cardiovascular diseases, further investigations scrutinizing gene-gene or gene-environment interactions on CRFs are required [21].

The results of this study do not contradict those of our previous reports. The present study shows that coffee consumption may decrease the number of CRF components among Mt5178C genotypic men, and previous studies have reported that coffee consumption decreases the risk of hypertension [10] and AGT [12]. On the other hand, the present study shows that coffee consumption may increase the number of CRF components among Mt5178A genotypic men, and a previous study reported that coffee consumption increases the risk of hyper-LDL cholesterolemia among Mt5178A genotypic men [11]. The present study shows a U-shaped association between the frequency of alcohol consumption and the clustering of CRFs among Mt5178C genotypic men. Our previous studies reported that daily alcohol consumption increases the risk of hypertension [13] or hyperuricemia [15], and decreases that of hyper-LDL cholesterolemia [14]. Therefore, the U-shaped association with the lowest risk of clustering of CRFs in occasional drinkers is acceptable. In view of the J-shaped relationship between alcohol consumption and coronary heart disease [22], the results of this study were interesting from a preventative medicine perspective.

CRFs overlap with components of metabolic syndrome (MtS). Although Balk et al. reported that coffee consumption is not associated with any components of MtS in Dutch men [23], several cross-sectional studies have shown that coffee consumption is inversely associated with MtS and the respective components of MtS in the Japanese population [24-26]. The results of this study showed that coffee consumption may decrease the clustering of CRFs in Mt5178C genotypic men, but not in Mt5178A genotypic men. Therefore, genetic factors, including Mt5178 C/A polymorphism, may modify the relationship between coffee consumption and components of MtS.

Even employing a systematic overview of 14 observational studies, the relationship between alcohol consumption and MtS remains controversial [27]. However, a large-scale prospective cohort study revealed that alcohol consumption is associated with independent components of MtS, comprising CRFs [28]. The results of this study suggested that daily drinking increases the clustering of CRFs for Mt5178C genotypic men, but not for Mt5178A genotypic men. Therefore, Mt5178 C/A other genetic polymorphisms most likely influence the relationship between alcohol consumption and components of MtS.

The mechanisms underlying the joint effects of Mt5178 C/A (ND2-237 Leu/Met) polymorphism and coffee or alcohol consumption on the clustering of CRFs remain unknown. They presumably depend on the amino-acid-related differences in response to ROS between ND2-237Leu and ND2-237Met. NADH dehydrogenase, namely complex I of the mitochondrial respiratory chain, is a major source of ROS production, and is a target of ROS [29]. Both observational and interventional studies have shown that coffee consumption exerts antioxidant potential [30,31]. Exertion of antioxidant activity of chlorogenic acid, caffeine and other natural compounds in coffee may be higher in men with ND2-237Leu than in those with ND2-237Met. Ethanol metabolism is directly associated with ROS production [32]. Alcohol-induced oxidative stress may be higher in men with ND2-237Leu than in those with ND2-237Met. The mechanisms responsible for the joint effects of Mt5178 C/A (ND2-237 Leu/Met) polymorphism and coffee or alcohol consumption on the clustering of CRFs remain a matter for further biochemical exploration.

The present study has several crucial limitations. First, the findings are limited by the small sample size, which was not sufficient to validate gene-environment interactions. Moreover, small sample size limited the definition of the clustering of CRFs as more than 2 or 3 CRF components. Second, the evaluation of habitual coffee consumption, which we used in previous studies [10-12], was based on the number of cups consumed per day, and this study lacked information regarding the method of coffee preparation. Third, the evaluation of habitual alcohol consumption, which we also used in previous studies [13-15], was based on the frequency of alcohol consumption. Forth, lack of information on waist circumference affected the evaluation of MtS as an outcome. By substituting BMI of ≥25 kg/m^2^ for waist circumference of ≥85 cm [33], we attempted to examine the interactions between Mt5178 C/A polymorphism and coffee consumption or alcohol consumption on the risk of MtS, but did not find any statistically significant associations in either genotype (data not shown). Fifth, we lacked information on dietary factors, which are reportedly related to the number of MtS components [34]. To overcome these limitations, a large-scale well-designed epidemiological study is necessary.

## Conclusion

Combined effects of longevity-associated Mt5178 C/A polymorphism and habitual coffee consumption or habitual alcohol consumption on the clustering of CRFs were observed in middle-aged Japanese men. For men with Mt5178C, who are susceptible to CVD, coffee consumption may decrease or daily alcohol consumption may increase the clustering of CRFs. Although further studies will be required to determine the individualized optimum for maximizing the risk reduction and minimizing the risk increase of habitual coffee or consumption for the clustering of CRF components, genetic information on Mt5178 C/A polymorphism will probably provide personalized prevention for CVD.

## Abbreviations

AGT: Abnormal glucose tolerance; BMI: Body mass index; CI: Confidence interval; CRFs: Cardiovascular risk factors; CVD: Cardiovascular disease; DBP: Diastolic blood pressure; FPG: Fasting plasma glucose; HDL: High-density lipoprotein; LDL: Low-density lipoprotein; MtS: Metabolic syndrome; Mt5178 C/A: Mitochondrial DNA 5178 cytosine/adenosine; ND2-237 Leu/Met: NADH dehydrogenase subunit-2 237 leucine/methionine; OR: Odds ratio; PCR: Polymerase chain reaction; ROS: Reactive oxygen species; SBP: Systolic blood pressure.

## Competing interests

The authors declare that they have no competing interests.

## Authors’ contributions

TI designed the study, analyzed the data, and drafted the manuscript; AK designed the study, carried out the epidemiological survey, carried out genotyping, analyzed the data, and drafted the manuscript; MI collected the samples; NM assisted with genotyping; KK and MY carried out the epidemiological survey; TO, HO, TS, HN and HH assisted in data analysis and helped with interpreting the results; YT designed the study and carried out the epidemiological survey. All authors have read and approved the final manuscript.
